# Ability of History Taking Alone to Identify Early Pregnancies Among Potential Measles Vaccinees

**DOI:** 10.1155/S1064744994000402

**Published:** 1994

**Authors:** Philip B. Mead

**Affiliations:** 1 Department of Obstetrics and Gynecology University of Vermont College of Medicine and Employee Health Service Medical Center Hospital of Vermont Burlington VT USA; 2 University Health Center Arnold 4 1 South Prospect Street Burlington VT 05401 USA

## Abstract

*Objective:* This study was undertaken to determine the ability of history taking alone, compared with pregnancy testing, to identify early pregnancies among potential female measles vaccinees.

*Methods:* As part of an institution-wide measles immunization program, 326 female health care workers (HCWs) who denied being pregnant underwent a urine pregnancy test prior to vaccination.

*Results:* Of the 326 women, 3 had positive pregnancy tests and were ultimately confirmed to be pregnant. Although all 3 women denied the possibility of pregnancy prior to testing, 2 had been unable to give an exact date for their last menstrual period (LMP).

*Conclusions:* In this group of 326 northern New England HCWs being immunized against measles during an outbreak, history taking alone failed to identify 3 pregnancies. If the inability to give an exact date of the LMP had been included as a discriminator, 2 additional pregnancies could have been suspected, but 1 pregnancy still would have gone undetected.


Infectious Diseases in Obstetrics and Gynecology 2:56–59 (1994)
© 1994 Wiley-Liss, Inc.

### Ability of History Taking Alone to Identify Early Pregnancies Among Potential Measles Vaccinees

**Philip B. Mead**

*Department of Obstetrics and Gynecology, University of Vermont College of Medicine, and Employee Health Service, Medical Center Hospital of Vermont, Burlington, VT*

ABSTRACT

***Objective:*** **This study was undertaken to determine the ability of history taking alone, compared with pregnancy testing, to identify early pregnancies among potential female measles vaccinees.**

***Methods:*** **As part of an institution-wide measles immunization program, 326 female health care workers (HCWs) who denied being pregnant underwent a urine pregnancy test prior to vaccination.**

***Results:*** **Of the 326 women, 3 had positive pregnancy tests and were ultimately confirmed to be pregnant. Although all 3 women denied the possibility of pregnancy prior to testing, 2 had been unable to give an exact date for their last menstrual period (LMP).**

***Conclusions:*** **In this group of 326 northern New England HCWs being immunized against measles during an outbreak, history taking alone failed to identify 3 pregnancies. If the inability to give an exact date of the LMP had been included as a discriminator, 2 additional pregnancies could have been suspected, but 1 pregnancy still would have gone undetected.** © 1994 Wiley-Liss, Inc.

KEY WORDS

Measles vaccination, pregnancy, pregnancy test

Although any possible effect of measles vaccine on fetal development is presently unknown, because it is a live-virus vaccine, its use is felt to be contraindicated in pregnant women.[1–6] Current guidelines state that identifying pregnancy in potential vaccinees by history alone is sufficient and pregnancy testing is not considered to be essential.[1,2,4,5] The literature, however, contains no studies of the effectiveness of history taking in determining whether potential vaccinees are pregnant.

During the winter of 1992–93, a measles outbreak occurred in Vermont, necessitating a mass immunization program at the Medical Center Hospital of Vermont, the state's 500-bed tertiary care teaching hospital. This program provided an opportunity to quantitate the ability of history taking alone, compared with pregnancy testing, to identify pregnant hospital employees who presented for measles immunization.

### SUBJECTS AND METHODS

When it became evident that the state was experiencing a measles outbreak, the hospital developed an institution-wide measles vaccination program. In the 2nd week of February 1993, all hospital employees, housestaff, and attending physicians born after 1957 were required to be evaluated at a central location for evidence of immunity to measles. Evidence of immunity was defined as birth before January 1, 1957, laboratory evidence of immunity to measles, documentation of physician-diagnosed measles, or documentation of the receipt of 2 doses of measles vaccine.[2] All of those without evidence of immunity were questioned with regard to contraindications to vaccination, including al-

Address correspondence/reprint requests to Dr. Philip B. Mead, University Health Center, Arnold 4, 1 South Prospect Street, Burlington, VT 05401.

Received February 18, 1994
Accepted June 13, 1994

lergy to eggs or neomycin, and altered immunocompetence. In addition, all women between the ages of 15 and 50 years were advised that pregnant women should not receive the measles vaccine and were asked if they were pregnant. Women who had no evidence of immunity, who had no contraindications to measles vaccination, and who stated they were not pregnant were asked to record the date of their last menstrual period (LMP) and sign the statement, "I do not believe I am pregnant and will wait until 3 months after vaccination before getting pregnant." Prior to being immunized, all women who denied being pregnant and who had signed the above statement had a urine pregnancy test (Abbott TestPack Plus, Abbott Laboratories, Abbott Park, IL) performed. If this test was negative, they were immunized.

### RESULTS

A total of 456 individuals had no evidence of immunity to measles, denied contraindications to measles vaccination including pregnancy, and agreed to be immunized. Of the 456, 128 were men and 328 were women. Of the 328 women, 2 refused pregnancy testing because of the history of either a total abdominal hysterectomy or a bilateral salpingo-oophorectomy. Of the 326 women who underwent a urine pregnancy test, 323 were negative and 3 were positive. These 3 cases are presented in more detail:

Case 1. A neurosurgery nurse stated she was not pregnant and signed the required statement, but could not remember the date of her LMP.

Case 2. An admitting clerk was seen on February 15. She stated she was not pregnant, signed the required statement, and listed her LMP as "? 1/19/93."

Case 3. A nutrition services worker was seen on February 15. She stated she was not pregnant, signed the required statement, and listed her LMP as "2/4/93."

All 3 pregnancies were subsequently confirmed.

### DISCUSSION

Because of the theoretical risk to the fetus of infection with a live-virus vaccine, women of childbearing age should receive measles, rubella, and mumps vaccines only if they are not pregnant.[1–6] How should one determine that a woman is not pregnant before she is vaccinated? The 3 most authoritative groups[2,4,5] concerning measles immunization of women recommend history taking alone as the means to exclude pregnancy.

In its October 1991 "Technical Bulletin on Immunization During Pregnancy," the American College of Obstetricians and Gynecologists states:

> Before a women is vaccinated, it should be determined whether she is pregnant. Because of the theoretical risk to the fetus of infection with live-virus vaccines, women of childbearing age should receive measles, rubella, and mumps vaccines only if they are not pregnant. Testing for pregnancy, however, is not essential. Reasonable precautions before giving these vaccines include asking women if they are pregnant, explaining the theoretical risks, and advising them not to become pregnant for 3 months after vaccination.[4]

The most recent recommendation of the Advisory Committee on Immunization Practices of the Centers for Disease Control regarding measles states:

> Live measles vaccine, when given as a component of MR (measles, rubella vaccine, live) or MMR (measles, mumps, and rubella vaccine, live), should not be given to women known to be pregnant or who are considering becoming pregnant within the next 3 months. Women who are given monovalent measles vaccine should not become pregnant for at least 30 days after vaccination. This precaution is based on the theoretical risk of fetal infection, although no evidence substantiates this theoretical risk. Considering the importance of protecting adolescents and young adults against measles, asking women if they are pregnant, excluding those who are, and explaining the theoretical risks to the others before vaccination are sufficient precautions.[2]

The 1991 Report of the Committee on Infectious Diseases of the American Academy of Pediatrics supports this approach as well.[5]

Despite the consistency of these recommendations, the sensitivity of history taking alone in identifying unsuspected pregnancies prior to immunization has not previously been reported. Our need to rapidly immunize a large number of predominantly female health care workers (HCWs) provided the opportunity to study this issue. Three (0.9%) of 326 women who, by carefully obtained and documented history, did not believe they were pregnant were found to be pregnant by urine pregnancy testing.

Could any systematic bias account for these findings? As all 3 women were subsequently confirmed as being pregnant, false-positive tests were not the explanation in this series. Most currently available office urine pregnancy tests have a specificity of nearly 100%.[7] Of greater concern is the fact that many employees may have known that a urine pregnancy test would be performed prior to receiving the measles immunization. It is possible that 1 or more of the 3 women with subsequently positive tests actually thought they were pregnant and were "testing the system." Such an explanation might account for case 1 and case 2 being uncertain of the date of their LMPs. In this series, if only those who denied being pregnant *and* listed a definite LMP had been considered not pregnant, history taking alone would have failed to identify only a single pregnancy. Adding the inability to give a precise date of the LMP as a discriminator may not be a practical strategy, however, as many women would presumably be unable to give such a date, limiting the specificity of this approach.

In practice, the use of pregnancy testing to corroborate a negative history is inconsistent. In the 1992–93 outbreak in Vermont, the Vermont Department of Health did not employ pregnancy testing in their measles immunization program while the hospital did. In a hospital-wide measles vaccination program reported from Hartford, CT, pregnancy tests were administered only to those employees with "uncertain pregnancy status,"[8] while a similar vaccination program from a hospital in Stony Brook, NY, reported in the same journal tested all female employees of childbearing age.[9]

A strategy to decrease the potential number of vaccinees, and the possibility of an undetected pregnancy, would be to provide prevaccination serologic screening. At our hospital, approximately 93% of tested HCWs are seropositive for measles antibody. Recent studies have shown that 90–94% of new or current medical personnel have antibody to measles virus.[10,11] In a population with a high prevalence of seropositivity, the use of a sensitive antibody screening test could identify the small subset of nonimmune individuals who need to be immunized against measles. Such a strategy has been shown to be cost effective.[12] In settings where rapid or widespread immunization is necessary, such as outbreaks or exposures or where return of those screened is not assured, prevaccination screening is problematic and is not recommended.[13]

The urine pregnancy test used in this program has a sensitivity of 50 mIU/ml of human chorionic gonadotropin and should identify pregnancy by the time of the 1st expected menstrual day with a sensitivity of 99.3%.[7] Thus, a small number of pregnant women with false-negative tests or very early gestations will not be detected despite urine pregnancy testing.

To summarize, history taking alone, as currently recommended, failed to identify 3 unsuspected pregnancies in a group of 326 HCWs. Two of these 3 women, despite believing themselves not to be pregnant, were unable to provide the exact date of their LMP. These observations were made in a cohort of northern New England HCWs in an outbreak setting. Whether they are generalizable to other populations, locations, or settings is unknown.

### REFERENCES

1. Centers for Disease Control: Measles prevention. MMWR 36(26):409–425, 1987.
2. Centers for Disease Control: Measles prevention: Recommendations of the Immunization Practices Advisory Committee. MMWR 38(S-9):1–18, 1989.
3. Centers for Disease Control: Update on adult immunization: Recommendations of the Immunization Practices Advisory Committee. MMWR 40(RR-12):1–50, 1991.
4. American College of Obstetricians and Gynecologists: Immunization during pregnancy. ACOG Technical Bulletin No. 160, October 1991.
5. Peter G (ed): Report of the Committee on Infectious Diseases. 22nd ed. Elk Grove Village, IL: American Academy of Pediatrics, p 318, 1991.
6. Anon.: Attenuvax. In: PDR 1994. 48th ed. Montvale, NJ: Medical Economics Co., p 1407, 1994.
7. Anon.: Abbott TestPack Plus. Package insert. Abbott Park, IL: Abbott Laboratories, Diagnostics Division, 1990.
8. Rank EL, Brettman L, Katz-Pollack H, DeHertogh D, Neville D: Chronology of a hospital-wide measles outbreak: Lessons learned and shared from an extraordinary week in late March 1989. Am J Infect Control 20:315–318, 1992.
9. Gurevich I, Barzarga RA, Cunha BA: Measles: Lessons from an outbreak. Am J Infect Control 20:319–325, 1992.

10. Willy ME, Koziol DE, Fleisher T, et al.: Measles immunity in a population of healthcare workers. Infect Control Hosp Epidemiol 15:12–17, 1994.
11. Wright LJ, Carlquist JF: Measles immunity in employees of a multihospital healthcare provider. Infect Control Hosp Epidemiol 15:8–11, 1994.
12. Subbarao EK, Amin S, Kumar ML: Prevaccination serologic screening for measles in health care workers. J Infect Dis 163:876–878, 1991.
13. Grabowsky M, Markowitz L: Serologic screening, mass immunization, and implications for immunization programs (letter). J Infect Dis 164:1237–1238, 1991.
